# Ultramarine, a Chromoprotein Acceptor for Förster Resonance Energy Transfer

**DOI:** 10.1371/journal.pone.0041028

**Published:** 2012-07-16

**Authors:** Anne Pettikiriarachchi, Lan Gong, Matthew A. Perugini, Rodney J. Devenish, Mark Prescott

**Affiliations:** 1 Department of Biochemistry and Molecular Biology, Monash University, Clayton Campus, Victoria, Australia; 2 ARC Centre of Excellence for Structural and Functional Microbial Genomics, Monash University, Clayton Campus, Victoria, Australia; 3 Department of Biochemistry, La Trobe Institute for Molecular Science, La Trobe University, Melbourne, Victoria, Australia; Stanford, United States of America

## Abstract

We have engineered a monomeric blue non-fluorescent chromoprotein called Ultramarine (fluorescence quantum yield, 0.001; ε _585__nm_, 64,000 M^−1^. cm^−1^) for use as a Förster resonance energy transfer acceptor for a number of different donor fluorescent proteins. We show its use for monitoring activation of caspase 3 in live cells using fluorescence lifetime imaging. Ultramarine has the potential to increase the number of cellular parameters that can be imaged simultaneously.

## Introduction

Pairs of fluorescent proteins (donor and acceptor) suitable for Förster resonance energy transfer (FRET) may be configured to monitor a myriad of cellular parameters including protein-protein interactions, post-translational modifications, flux of metabolic intermediates and other biochemical events that constitute the molecular networks of the cell [1]. However, experimental design and subsequent data analysis is often constrained by technical issues including spectral overlap between donor and acceptor emissions, and direct acceptor excitation. There have been a number of recent reports describing new FRET pairs (fluorescent donor and acceptor) optimized for use together with minimal spectral bleed-through [2], [3]. The use of a non-fluorescent or dark acceptor is one approach to eliminating such complications, and facilitating multi-parameter imaging in single cells. In such cases fluorescence lifetime imaging microscopy can be used to follow FRET between the donor and dark acceptor [4].

## Results

**Figure 1:**
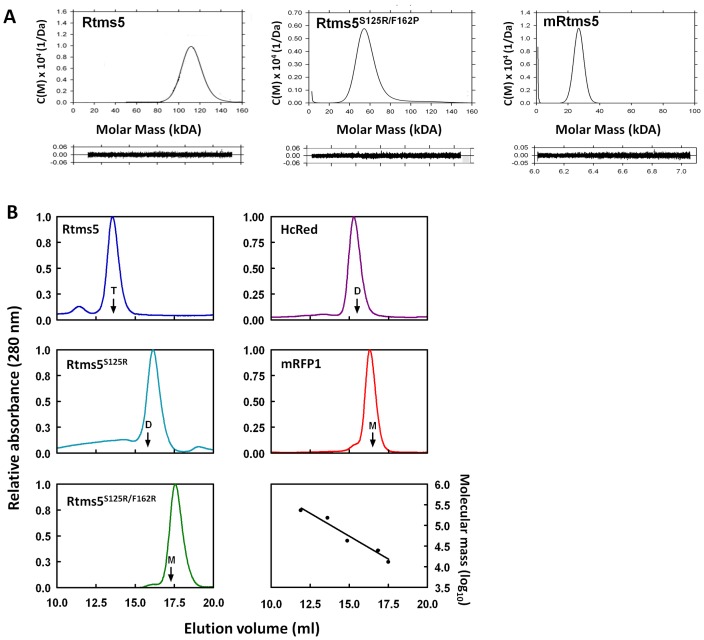
Analysis of Rtms5 and its variants by analytical ultracentrifugation and size-exclusion chromatography. (A). Samples of Rtms5 (tetramer), Rtms5^S125RF162P^ (dimer) and mRtms5 (monomer) purified by Ni-NTA chromatography at 1 mg.ml^−1^ were subjected to analytical ultracentrifugation. Residuals for each of the plots are shown. Two additional substitutions were introduced to mRtms5 to produce the faster maturing Ultramarine with improved extinction coefficient and lower Ф_F_. (B). Proteins (100 µl of 1 mg.ml^−1^) purified by Ni-NTA chromatography were subjected to gel filtration chromatography on a Superdex 10/300 S200 column. The labeled arrows indicate the predicted elution volume for tetramers (T), dimers (D) and monomers (M) based on calibration of the column with native molecular weight standards. Results for Rtms5 (tetramer), HcRed (dimer) and mRFP (monomer) are included for comparison. The results of AUC experiments indicate that Rtms5 and HcRed are tetramers and dimmers, respectively [23]. mRFP1 is reported to be a monomer [9]. A small proportion of wildtype Rtms5 eluted as oligomers larger than tetramers.

We sought to engineer a genetically encoded dark acceptor that could be used with a range of donor fluorescent proteins (FPs). Naturally occurring non-fluorescent chromoproteins are suitable candidates for dark acceptors as they typically exhibit high extinction coefficients, broad absorption spectra and very low fluorescence emission efficiencies [5], [6], [7]. However, the chromoproteins isolated to date are obligate tetramers making them unsuitable for use as fusion tags [8]. The chromoprotein, Rtms5, isolated from the reef building coral *Montipora efflorescens* [5] has a high extinction coefficient (λ_max_, 592 nm; ε = 80,000 M^−1^cm^−1^) and is very weakly fluorescent (fluorescence quantum yield (Φ_F_)_,_ 0.004; **λ** ^Em^_ max_, 636 nm) (**Table 1**). Analytical ultracentrifugation (AUC) analysis indicates that Rtms5 behaves as a tetramer in solution even at relatively low protein concentrations (0.1 mg/ml) (**Fig. 1A**). The X-ray crystal structure of Rtms5 revealed that the quaternary structure is stabilized by interactions involving the side-chains of serine 125 (A/B interface) and phenylalanine 162 (A/C interface) [5] on neighboring protomers. We reasoned that substituting these amino acids with arginine would facilitate the formation of Rtms5 monomers.

Using site-directed mutagenesis variants Rtms5^S125R^, Rtms5^F162R^ and Rtms5^S125R/F162R^ were generated and the properties of these proteins expressed in bacteria investigated. Rtms5^S125R^ is an intensely blue colored protein which was determined by gel filtration chromatography to behave predominantly as a dimer (**Fig. 1B** **;** **Table 1**). Rtms5^F162R^ and Rtms5^S125R/F162R^ when expressed in bacteria led to colonies having little or no blue color suggesting a defect in formation of the chromophore. The results of gel filtration chromatography experiments indicated that Rtms5^F162R^ and Rtms5^S125R/F162R^ proteins behaved predominantly as a dimer and a monomer, respectively (**Fig. 1B**).

We next investigated the possibility that other substitutions at position 162 might promote formation of a monomer with a functional chromophore. Random mutagenesis of the codon for the amino acid at position 162 using a template encoding Rtms5^S125R/F162R^ resulted in a number of variants each with a functional chromophore, but which all eluted from gel filtration columns as dimers (data not shown). One variant, Rtms5^S125R/F162P^ which showed relatively fast maturation of the chromophore (t_0.5_ = 1.8 h at 28°C) (**Table 1**) was confirmed to behave as a dimer by AUC (**Fig. 1A**). Rtms5^S125R/F162P^ was expressed as a tandem dimer linked via a 13 amino acid polypeptide (t-Rtms5^S125R/F162P^). This tandem dimer was found to have a molecular extinction coefficient almost twice that of Rtms5^S125R/F162P^ (Table 1) and may find use in the pseudo-monomer format [9] as a dark acceptor in applications where the size of the tag is not a limitation and increased FRET efficiency is required.

**Figure 2:**
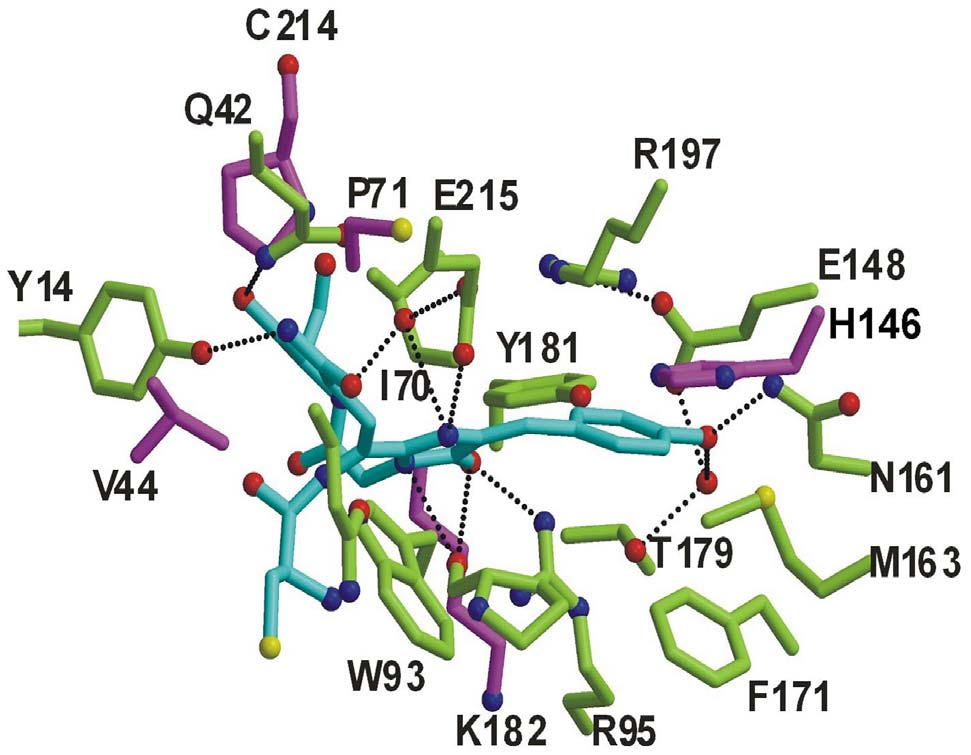
Rtms5 chromophore contacts and location of some substitutions. The Rtms5 non-coplanar *trans* chromophore (blue) and contacting residues (green and purple) are shown. The position of some of the amino acids identified in the random screen (purple) which when substituted were shown to improve chromophore maturation of Rtms5–7 are located close to residues contacting the chromophore.

We next used a random mutagenesis expression-screen to isolate monomers of Rtms5 that contained a functional chromophore. The initial input to the screen, a colorless variant Rtms5^S125R/F162R/L127R/A164R/L174T/Y192R/Y194R^ (designated Rtms5–7) included five additional protomer interface substitutions compared to Rtms5^S125R/F162R^. This strategy was adopted to reduce the probability of false positives arising from reversion to arginine at positions 162 or 125 and formation of a functional chromophore due to dimerisation or tetramerisation. Rtms5–7 was subjected to two rounds of PCR-based random mutagenesis. Out of approximately 50,000 colonies 100 showed appreciable blue color on expression plates. The single substitutions V44A, P71A and C214Y were found individually in three of the most intensely colored variants, and accordingly were individually introduced into Rtms5^S125R/F162R^. The side-chains of these three amino acid substitutions, and those of 27 others identified in the screen were either close to the chromophore or contacting the chromophore (**Fig. 2**). Of the three variants Rtms5^S125R/F162R/V44A^ displayed the most intense blue color and analysis by AUC confirmed it to behave as a monomer at 1 mg/ml (**Fig. 1A**). This variant was designated mRtms5. The effects of introducing to mRtms5 further amino acid substitutions were investigated based on results from our PCR-based random mutagenesis screens, and data emerging from other studies. The substitution H146N was identified in the first round of random mutagenesis as one which resulted in colonies with appreciable blue colour. The substitution L127T made on the A/B interface was introduced to improve the stability of monomers as one other study had reported the benefit of substitutions at this position [10]. Introduction of the substitutions H146N and L127T to mRtms5 resulted in a variant, hereafter referred to as Ultramarine, with an increased extinction coefficient and decreased Φ_F_ compared to mRtms5 (**Table 1**). The absorption spectra of rapidly purified proteins were determined and used to follow chromophore maturation at both 28°C and 37°C. Ultramarine was calculated to have maturation t_0.5_∼1.8 h at 37°C (**Table 1**) which is comparable to that of Rtms5 and some commonly used FPs such as cpT-Sapphire and the DsRed dimer2 [9].

**Table 1:** Some properties of Rtms5 variants.

Protein	λ_max_^Abs^ (nm)	λ_max_^Em^ (nm)	ε at λ_max_^Abs^(M^−1^cm^−1^)	ΦF		t_0.5_ maturation	
						28°C	37°C
Rtms5	592	636	80 000	0.004	Tetramer^1^	<1 h	<1 h
Rtms5 ^S125R F162P^	590	633	50 900	0.002	Dimer^1^	1.8 h	ND
t-Rtms5 ^S125R F162P^	591	635	107 800	0.004	Dimer^2^	<1 h	ND
mRtms5	588	633	54 100	0.005	Monomer^1^	ND	ND
Ultramarine	586	626	64 000	0.001	Monomer^3^	3.6 h	1.8 h

Properties were determined for purified protein in 20 mM Tris-HCl, pH 8.0 and 300 mM NaCl.

1 Determined by AUC.

2 Determined by SDS-PAGE for non-boiled samples (pseudo-native PAGE).

3 Determined by size-exclusion gel filtration chromatography.

ND: not determined.

**Figure 3:**
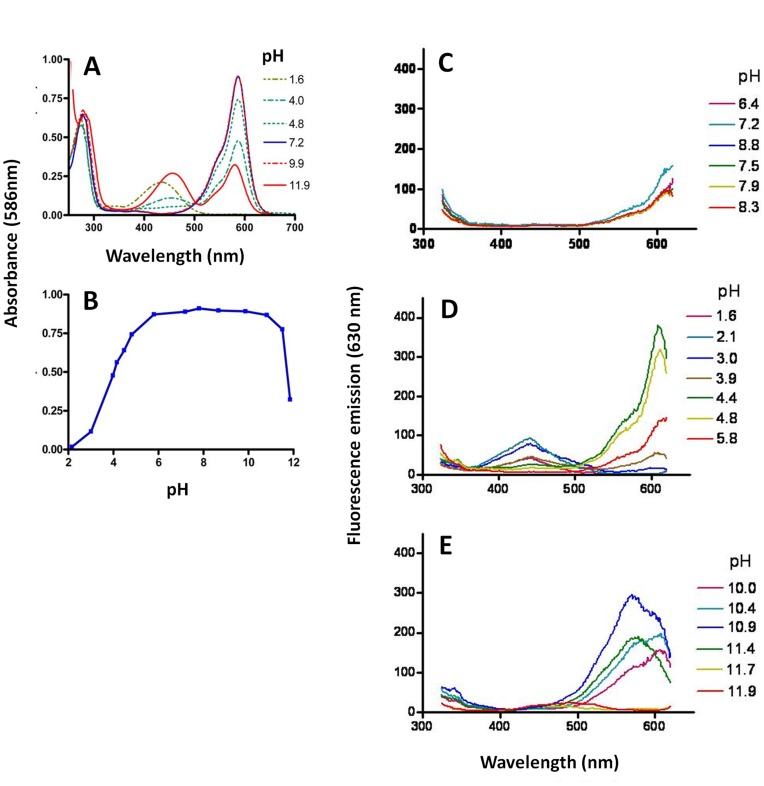
The effect of pH on some optical properties of Ultramarine. (A). The absorbance spectrum was determined for Ultramarine diluted in buffers of different pH and constant ionic strength. The species absorbing at ∼450 nm and ∼590 nm correspond to the neutral and anionic form of the chromophore, respectively. (B). Absorbance at 586 nm was determined at different pH. The species absorbing at 586 nm corresponds to the anionic chromophore and has an estimated pK_a_ of 4.1. Excitation spectra were obtained for Ultramarine (λ_Em_, 630 nm). Excitation scans are grouped into what are considered to be physiological (C), low (D) and high (E) pH ranges.

The effect of pH on some optical properties of Ultramarine was determined. Absorbance at 586 nm was stable over the pH range 6.0–10.5 (**Fig. 3** **A, B**) whilst the emission (**λ** ^Em^_max_, 630 nm) at different excitation wavelengths remained very low over the physiological pH range 6.4–8.3 (**Fig. 3** **C, D, E**). These results indicate that Ultramarine maintains the desirable acceptor properties of low Ф and high extinction coefficient over a wide range of pH.

**Figure 4:**
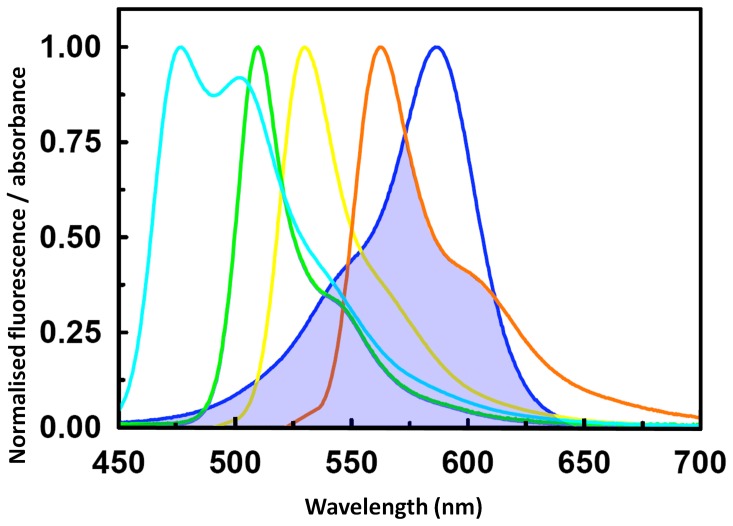
Overlap of Ultramarine absorbance with fluorescence emission of some donor fluorescent proteins. The absorbance spectrum for Ultramarine (blue) is shown overlayed with the fluorescence emission spectra of selected donor FPs (Cerulean, cyan; cpT-sapphire and Sapphire, green; EYFP, yellow and mKO, orange). The overlap between Ultramarine absorbance and donor fluorescence emission is highlighted by light blue fill.

The broad absorption spectrum and high extinction coefficient (64,000 M^−1^cm^−1^; 586 nm) suggested to us that Ultramarine would be suitable as a dark acceptor for the emissions of a range of donor FPs. The overlap between the emission spectra of five different FPs and the absorbance spectra of Ultramarine is shown (**Fig. 4**). *R*_0_ values for each donor paired with Ultramarine were calculated and ranged between 44.6 Å to 59.2 Å (**Table 2** **)**. Ultramarine was expressed fused individually to each of five different donor FPs, mKO [11], EYFP [11], [12], Cerulean [13], cpT-Sapphire [14] and Sapphire [14] via a short polypeptide containing the caspase 3 protease recognition sequence DEVD (C3). Caspase 3 is a member of a family of proteases central to the cell death machinery involved in apoptosis [15]. The fold-increase in donor fluorescence upon incubation of purified fusion proteins with protease ranged from 1.8–3.4 indicative of significant FRET with Ultramarine (**Table 2**).

**Figure 5:**
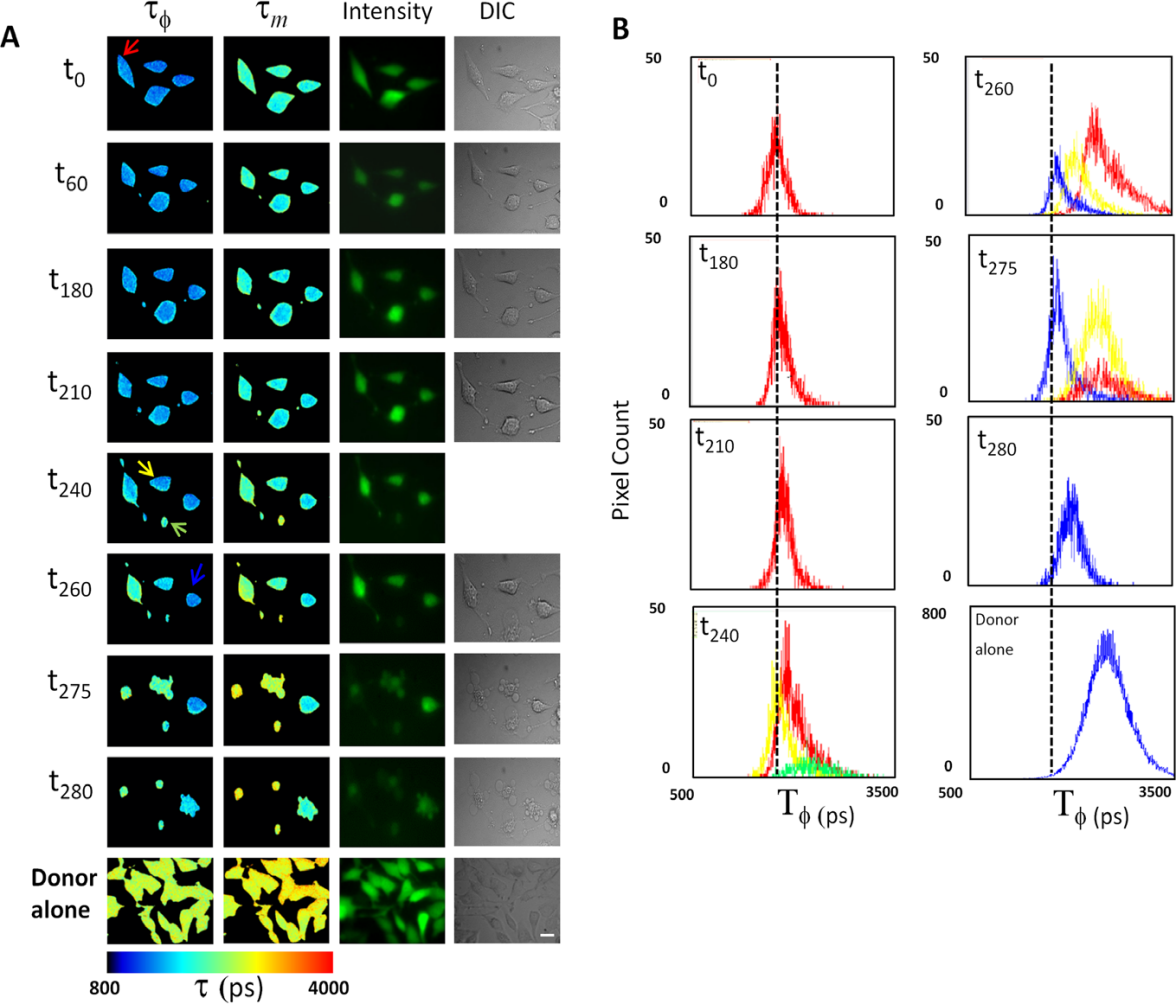
Using FLIM to monitor caspase 3 activation in live cells expressing Ultramarine^hC3^cpT-Sapphire. (A). Color maps of fluorescence lifetime data (Phase, τ_ϕ_ and modulation, τ_m_) are shown for HeLa cells expressing Ultramarine^hC3^cpT-Sapphire or cpT-Sapphire. For this experiements an Ultramarine expression cassette (Ultramarine^h^) codon optimised for use in human cells was used. Cells were imaged in a time series before (t_0_) and after addition of staurosporine (t_60_–t_280_) to initiate activation of Caspase 3. Fluorescence intensity and DIC images are shown. Lifetime images are shown for cells expressing cpT-Sapphire (donor alone). Colored arrows highlight individual cells for which lifetime distributions are plotted in (B). Lifetime images are shown for cells expressing cpT-Sapphire (donor alone). Time of incubation (mins) after addition of STS is shown at left. Scale bar  = 10 µm (B). The lifetime distributions for τ_ϕ_ are plotted for selected cells at different times of incubation. The line color corresponds to the cell highlighted by the same color arrow in A. The dashed line (centered on 1.6 ns) serves as a reference to highlight a progressive shift towards longer lifetimes. The lifetime distribution for the donor alone is plotted for all cells in the field of view.

The use of Ultramarine in cell culture was investigated in HeLa cells expressing the Ultramarine^hC3^cpT-Sapphire fusion protein (**Fig. 5**). The activation of caspase 3 protease activity induced by incubating cells with 2 µM stauropsorine (STS), and the resultant change in FRET efficiency followed in live single cells by measuring the donor fluorescence lifetime using FLIM (**Fig. 5A**). STS is a frequently used agent for the rapid and complete induction of apoptosis that involves caspase 3 activation [16]. The donor emission was imaged at selected time points and the lifetime images (τ_Ф_ and τ_m_) calculated. The corresponding fluorescence lifetime, fluorescence intensity and DIC images representing 4 individual cells in a field of view are shown. The lifetime (τ_Ф_) distributions are shown in histogram form for each cell (**Fig. 5B**) at selected time points color-coded according to the arrow highlighting the particular cell (**Fig. 5A**). The color maps show that the average τ_Ф_ and τ_m_ lifetimes for each individual cell at t_o_ change relatively little for the first 180 mins after addition of STS. Nevertheless, the intensity and DIC images show evidence that the cells have started to undergo alterations in morphology characterized by rounding of the cells. At different incubation times, depending on the individual cell, fluorescence lifetimes were observed to increase (**Fig. 5A**). τ_Ф_ for one cell (red arrow) shifted progressively to higher values. At t_260_ the increase in lifetime was significant, corresponding to a reduction in FRET and cleavage of the linker joining the components of Ultramarine^hC3^cpT-Sapphire by activated caspase 3. Blebbing of the plasma membrane, a hall mark of apoptosis is evident in the DIC image at t_275_. The activation of caspase 3 at different time points was observed for each cell (yellow and blue arrow). One cell (green arrow) underwent significant caspase 3 activation and membrane blebbing between t_260_ and t_275_. The fluorescence lifetimes were determined for cells expressing the donor alone (cpT-Sapphire) and are shown as color maps (**Fig. 5A**) and τ_Ф_ distributions (**Fig. 5B**). Collectively, these results show that Ultramarine can be used as a dark acceptor to monitor events in live cells.

**Table 2:** Increases in fluorescence intensity for different donor fluorescent proteins fused to Ultramarine after protease cleavage.

Fusion Protein	Fold Increase in donor emission (at λ^Em^_max_)^1^	R_0_ (Å)^2^
Ultramarine^C3^EYFP	3.4	52.0
Ultramarine^C3^mKO	3.3	59.2
Ultramarine^C3^cpT-Sapphire	3.2	46.8
Ultramarine^C3^Sapphire	2.9	45.9
Ultramarine^C3^Cerulean	1.8	44.6
ECFPC3EYFP	1.5	–

1 Increase in fluorescence emission was monitored until an endpoint was reached. Complete cleavage of the polypeptide linker was confirmed by subjecting endpoint samples to analysis SDS-PAGE.

2 R_0_ values were calculated according to [29].

For comparison data is included for the fusion protein ECFP^C3^EYFP, a probe reported for measuring caspase 3 protease activation in cells [30].

REACh, a dark yellow FP has been reported as having been optimized for use as an acceptor for GFP using FLIM [17]. By virtue of its extended conjugation chromophore conjugation system [5], Ultramarine has a broad red-shifted absorption spectrum compared to REACh, and therefore has significant spectral overlap with the emission of donors other than green emitting FPs (**Fig. 4**) and may be suitable as an acceptor to FPs with red emissions. Ultramarine has the potential to be used as an acceptor for multiple different donors for FRET in the same cell (providing the different donor emissions can be isolated from one another) and to increase the number of cellular parameters than can be imaged simultaneously.

## Materials and Methods

### Construction of Expression Vectors and Mutagenesis

An expression cassette encoding Rtms5 flanked by a 5′*Bam*HI site and 3′ by nested *Bcl*I/*Not*I sites was amplified by PCR using the primer pair Rtms515up/Rtms5Bcldo (Table 3) and pRSET-B:Rtms5 as template [18]. The PCR product was digested with *Bam*HI and *Not*I and ligated into the *Bam*HI/*Not*I expression site of pQE9N to produce pQE9N:Rtms5. This vector is the starting point for all subsequent mutagenesis experiments and expression screens, and encodes Rtms5 with an N-terminal hexahistidine tag used for purification of expressed proteins. pQE9N is a derivative of pQE9 (Qiagen) modified to contain a *Not*I site in the multiple-cloning site [5].

**Table 3:** Some oligonucleotide primer sequences used in this study.

Primer	Sequence 5′–3′
Rtms5up1	TAGGATCCAAAACAATGAGTGTGATCGCTACAC
Rtms5Bcldo	ATAGTTTAGCGGCCGCTCAGTGATCAGAGGCGACCACAGGTT TGC
Rtms513up2	TCGGATCCAGGTACTGGTTCTACTGGTTCTGGTTCTTCTATGAGTGTGATCGCTACAC
EYFPup	TAGGATCCTTCAGGACTACGATCGGGCGGCGACGAAGTGGACGGCGGCTCGAACTCGAGATCTATGTCTAAAGGTGAAG
EYFPdo	TTAGCGGCCGCTCAGTGATCAGTTACTTGTACAGCTCGTCCATG

Nucleotide changes specifying individual amino acid substitutions in an Rtms5 expression cassette were introduced using QuickChange site-directed mutagenesis (Stratagene, USA) using pQE9N:Rtms5 as template to produce the expression vectors pQE9N:Rtms5^S125R^, pQE9N:Rtms5^F162R^ and pQE9N:Rtms5^S125R/F162R^.

The amino acid at position 162 of Rtms5 was randomised by PCR mutagenesis using a single oligonucleotide primer degenerate for nucleotides encoding codon at position 162 and pQE9N:Rtms5^S125R/F162R^ and template [19]. An expression screen resulted in the isolation of pQE9N:Rtms5^F125R/F162P^.

An expression cassette encoding a tandem-dimer of Rtms5^S125R/F162P^ was constructed as follows. An expression cassette encoding Rtms5^S125R/F162P^ flanked 5′ by a *Bam*HI site and 3′ by a sequence encoding a 13 amino acid polypeptide linker (SDPGTGSTGSGSS) followed by a *Bcl*I site was amplified by PCR using pQE9N:Rtms5^S125R/F162P^ as template and primer pair Rtms513up2/Rtms5Bcldo. The PCR product was ligated into the *Bam*HI site of pQE9N:Rtms5^S125R/F162P^ to produce pQE9:t-Rtms5^S125R F162P^ encoding an expression cassette for two Rtms5^S125R/F162P^ polypeptides fuesed in tandem.

A DNA template encoding the monomeric colorless variant, Rtms5–7 (Rtms5^S125R/F162R/L127R/A164R/L174T/Y192R/Y194R^) used for the purposes of random mutagenesis and isolation of monomer variants was produced by the sequential introduction of individual codon changes using QuickChange mutagenesis and Rtms5^S125R/F162R^ as template. Random mutagenesis of the Rtms5–7 expression cassette was performed using the Genemorph Random Mutagenesis Kit (Stratagene, USA) and Rtms5^S125R/F162R/L127R/A164R/L174T/Y192R/Y194R^ template. The manufacturer’s protocol was adjusted to achieve a mutational frequency of 0–3 mutations/kb.

Expression cassettes encoding Ultramarine (Rtms5^S125R/F162R/V44A/L127T/H146N^) fused to the N-terminus of EYFP via a caspase 3 protease recognition site were constructed as follows. An expression cassette encoding EYFP with an N-terminal caspase 3 (C3) recognition motif (DEVD) and flanked by 5′ *Bam*HI and 3′ *Not*I sites was amplified by PCR using primer pair EYFPup and EYFPdo (Table 3). In this study a variant of EYFP enhanced for use in yeast was used [20]. The PCR product was digested with *Bam*HI and *Not*I and ligated into the *Bcl*I*/Not*I sites of pQE9N:Ultramarine to produce pQE9N:Ultramarine^C3^EYFP. In order to facilitate subsequent cloning of expression cassettes for alternative donor proteins, the primer EYFPup encodes a *Bgl*II site between the sequence encoding the C3 recognition motif and the FP. The *Bgl*II/*Not*I fragment in pQE9N:Ultramarine^C3^EYFP encoding EYFP was exchanged for expression cassettes flanked 5′ and 3′ by *Bam*HI and *Not*I sites, respectively and encoding mKO [21], cpT-Sapphire, Sapphire [14] or Cerulean [22] to produce vectors pQE9N:Ultramarine^C3^mKO, pQE9N:Ultramarine^C3^cpT-Sapphire, pQE9N:Ultramarine^C3^Sapphire and pQE9N:Ultramarine^C3^cerulean.

An expression cassette encoding Ultramarine codon optimised for expression in human cells (Utramarine^hC3^), and flanked by a 5′ *Sma*I site followed by a sequence encoding a caspase 3 recognition motif and nested *Bgl*II*/Not*I sites was chemically synthesized and supplied ligated into pUC57 as pUC57:Ultramarine^hC3^ (Genscript, USA). An expression cassette encoding cpT-Sapphire flanked by *Bam*HI/*Not*I sites was released from pQE9N:Ultramarine^C3^cpT-Sapphire and ligated into the *Bgl*II*/Not*I of pUC57:Ultramarine^hC3^ to produce pUC57:Ultramarine^hC3^cpT-Sapphire. A *Sma*I*/Not*I fragment encoding Ultramarine-^C3^-CpT-Sapphire was released from pUC57:Ultramarine^hC3^cpT-Sapphire and ligated into the *Sma*I*/Not*I expression site of the mammalian expression vector pCI-neo (Promega) containing a mitochondrial targeting sequence from subunit VIII of cytochrome *c* oxidase [23] to produce pCI-Neo:Ultramarine^hC3^cpT-Sapphire. The *Bam*H1/*Not*I fragment from pCI-Neo:Ultramarine^hC3^cpT-Sapphire was exchanged for the *Bam*H1/*Not*I fragment from pQE9N:cpT-Sapphire to produce pCI-Neo:cpT-Sapphire.

### Protein Expression and Purification

For the routine production of recombinant proteins *E. coli* (BL21-DE3) cells freshly transformed with pQE9N expression vectors were induced and protein purified using Ni-NTA chromatography as described [24]. For expression screens transformation mixtures were plated onto solid selective media supplemented with isopropyl β-D-1-thiogalactopyranoside (IPTG; 0.1 mM) to induce protein expression. Plates were scored according to the appearance of colonies and the intensity of blue colour 24 h after plating.

For chromophore maturation studies tight control of protein expression was obtained by using the *E. coli* host strain M15 [pREP4] (Qiagen). Cells freshly transformed with the appropriate pQE9N expression vector were inoculated into 50 mL LB medium with ampicillin and kanamycin, and incubated with shaking at 37°C overnight. An aliquot of the overnight culture was diluted into 250 ml fresh growth medium to an OD_600_, 0.71 and incubated for a further 2 h after which expression was induced by the addition of 0.2 mM IPTG. After 6 h incubation cells were harvested, chilled on ice, lysed using Y-PER Yeast Protein Extraction Reagent (Pierce) and protein purified at 4°C using Ni-NTA chromatography. Time taken from cell harvest to analysis was ∼40 min. This approach minimized chromophore maturation during cell harvesting and protein purification.

### Spectroscopy

Purified protein underwent exhaustive dialysis against 20 mM Tris-HCl, pH 8.0, 300 mM NaCl and concentrated using a centrifugal ultrafiltration device (Millipore Corp., MWCO 10,000). Fluorescence spectra and absorbance spectra were determined using a Varian Eclipse fluorescence spectrophotometer and a Varian Cary 50 spectrophotometer (Melbourne, Australia), respectively. Fluorescence quantum yields (Φ_F_) were determined for protein samples in 20 mM Tris-HCl, pH 8.0 and 300 mM NaCl at 24°C using a solution of cresyl violet (Φ_F_, 0.54) in methanol as a standard. Optical characteristics of proteins at different pH were determined as described [25].

### Gel Filtration Chromatography

Protein samples were analysed for size by gel filtration chromatography [5], [26] using an AKTA FPLC system (Amersham). Protein samples (100 µl of 1 mg/ml) were applied at a flow rate of 1 ml/min to a Superdex S200 10/30 column equilibrated with 20 mM Tris-HCl, pH 8.0, 300 mM NaCl and eluates monitored at 280 nm. The column was calibrated using Healthcare Calibration Kits (Gel filtration LMW Calibration Kit and Gel filtration HMW Calibration Kit) containing the following proteins ferritin (440 kDa), catalase (232 kDa), aldolase (158 kDa), ovalbumin (43 kDa), chymotrypsinogen A (25 kDa) and ribonuclease A (13.7 kDa).

### Analytical Ultracentrifugation

Sedimentation velocity experiments were conducted using a Beckman model XL-I analytical ultracentrifuge at a temperature of 20°C. Samples of Rtms5, Rtms5^S125R F162P^ and mRtms5 dissolved in 300 mM NaCl, 20 mM Tris pH 8.0 were loaded into a conventional double-sector quartz cell and mounted in a Beckman 4-hole An-60 Ti rotor. 380 µl of sample (0.1 mg/ml and 1.0 mg/ml) and 400 µl of reference solution were centrifuged at a rotor speed of 40,000 rpm, and the data collected at a single wavelength (485 or 580 nm) in continuous mode, using a time interval of 300 s and a step-size of 0.003 cm without averaging. Solvent density (1.011 g/ml at 20°C) and viscosity (1.035 cp), as well as estimates of the partial specific volume of Rtms5 (0.7265 ml/g), Rtms5^F162P/S125R^ (0.7265 ml/g) and mRtms5 (0.7248 ml/g) were computed using the program SESDNTERP [27]. Sedimentation velocity data at multiple time points were fitted either to a single discrete species model or a continuous size-distribution model [28] using the program SEDFIT, which is available at www.analyticalultracentrifugation.com.

### Protease Cleavage of Fusion Proteins *in vitro*

500 µl of 0.52 mg/ml purified protein in 20 mM Hepes pH 7.5, 10 mM KCl, 2.5 mM MgCl_2_, 1 mM EDTA, 1 mM EGTA and 1 mM DTT for each of the different Ultramarine-^C3^-FP fusion proteins was incubated with protease at 37°C. Fluorescence emission of the donor FP was monitored at regular time intervals until an endpoint was reached. The fold-increase in donor emission was defined as: donor fluorescence intensity at endpoint/donor fluorescence before incubation with protease. Samples of reactions at the endpoint were subjected to analysis by SDS-PAGE to confirm efficient cleavage of the polypeptide linker between the donor and Ultramarine.

### Mammalian Cell Expression

HeLa cells maintained at 37°C and 5% CO_2_ in Dulbecco’s modified Eagle’s medium supplemented with 10% (v/v) fetal calf serum were transfected in glass-bottomed Fluorodishes (World Precision Instruments Inc) with pCI-Neo:Ultramarine^hC3^cpT-Sapphire using FuGene reagent (Promega). At 48 h post transfection cells were imaged on a heated stage maintained at 37°C (see below). Caspase 3 was activated in cells by the addition of STS (Sigma-Aldrich) to a final concentration of 2 µM. HeLa cells were obtained from the American Type Culture Collection (ATCC; USA).

FLIM was performed on a frequency domain system supplied by La Vision BioTec (Bielefeld, Germany) coupled to an Olympus IX81 microscope. Illumination was provided by a modulated (80 MHz) laser diode (410 nm) fibre-coupled into the back illumination port of the microscope. Image data was acquired using a Picostar HR full-field detector was coupled to the side port of the IX81 consisting of a gated optical intensifier driven by a high rate imager and CCD camera. Two signal generators were implemented in a master/slave configuration to modulate the light source and the Picostar HR with the appropriate phase delays. Illumination was routed through a 403 nm beam splitter and fluorescence emission collected through a 510–550 nm barrier filter. Image data for 10 phase steps over 2π were routinely acquired for the calculation of lifetimes (τ_Ф_ and τ_m_). The system was controlled and image data analysed using ImSpector software (Version 2.3.6; LaVision Biotec). The system was calibrated for fluorescence lifetime using an alkaline solution of fluorescein assuming a fluorescence lifetime of 4.1 ns. Measurements were carried out at 37°C. The imaging platform system was equipped with the components of an Olympus FV500 confocal laser scanning system coupled through the remaining side-port of the IX81. DIC images were acquired upon illumination with 543 nm laser light.
